# A Hierarchical Generative Framework of Language Processing: Linking Language Perception, Interpretation, and Production Abnormalities in Schizophrenia

**DOI:** 10.3389/fnhum.2015.00643

**Published:** 2015-11-27

**Authors:** Meredith Brown, Gina R. Kuperberg

**Affiliations:** ^1^Department of Psychiatry–Athinoula A. Martinos Center for Biomedical Imaging, Massachusetts General Hospital, CharlestownMA, USA; ^2^Department of Psychology, Tufts University, MedfordMA, USA

**Keywords:** schizophrenia, language, generative models, speech perception, thought disorder, auditory verbal hallucinations

## Abstract

Language and thought dysfunction are central to the schizophrenia syndrome. They are evident in the major symptoms of psychosis itself, particularly as disorganized language output (positive thought disorder) and auditory verbal hallucinations (AVHs), and they also manifest as abnormalities in both high-level semantic and contextual processing and low-level perception. However, the literatures characterizing these abnormalities have largely been separate and have sometimes provided mutually exclusive accounts of aberrant language in schizophrenia. In this review, we propose that recent generative probabilistic frameworks of language processing can provide crucial insights that link these four lines of research. We first outline neural and cognitive evidence that real-time language comprehension and production normally involve internal generative circuits that propagate probabilistic predictions to perceptual cortices — predictions that are incrementally updated based on prediction error signals as new inputs are encountered. We then explain how disruptions to these circuits may compromise communicative abilities in schizophrenia by reducing the efficiency and robustness of both high-level language processing and low-level speech perception. We also argue that such disruptions may contribute to the phenomenology of thought-disordered speech and false perceptual inferences in the language system (i.e., AVHs). This perspective suggests a number of productive avenues for future research that may elucidate not only the mechanisms of language abnormalities in schizophrenia, but also promising directions for cognitive rehabilitation.

## Introduction

Schizophrenia is a complex and heterogeneous brain disorder affecting approximately one in 100 adults worldwide (Regier et al., 1993). In addition to its positive, negative, and disorganization symptoms, it is characterized by abnormalities across multiple domains of cognition (e.g., Kuperberg and Heckers, 2000; Bowie and Harvey, 2006; Carter et al., 2008), as well as by abnormalities in lower-level perception (e.g., Green et al., 2009; Javitt and Freedman, 2015). Language plays a central role in all these aspects of schizophrenia. First, abnormalities in language manifest in the major symptoms of psychosis itself, particularly in disorganized language output (positive thought disorder) and auditory verbal hallucinations (AVHs). Indeed, some of these clinical language disturbances are evident in individuals at high risk for developing the disorder (Klosterkötter et al., 2001; Solomon et al., 2011; Thompson et al., 2011). Second, language dysfunction is one of the core cognitive sequelae of schizophrenia, with verbal abilities often compromised relative to other cognitive domains in both patients with schizophrenia (e.g., Wexler et al., 1998; Wood et al., 2007), and in people at risk for developing the disorder (Fuller et al., 2002; Lencz et al., 2006; Pukrop et al., 2007; Simon et al., 2007; Fusar-Poli et al., 2012; Koutsouleris et al., 2012). Third, patients’ difficulties with encoding, representing, and categorizing auditory stimuli also extend to the perceptual processing of speech (e.g., Cienfuegos et al., 1999; Kasai et al., 2002; Fisher et al., 2008). Finally, language abnormalities have been linked to functional and social impairments (e.g., Holshausen et al., 2014).

Given the importance of language to so many aspects of schizophrenia, it is not surprising that language has long been a focal point of clinical and scientific interest in the disorder (Bleuler, 1950). Nonetheless, the literature on language in schizophrenia to date has been somewhat disjointed. One line of research has focused on the incoherent speech associated with thought disorder — the best known and most obvious clinical manifestation of language dysfunction in schizophrenia (Bleuler, 1950; Andreasen, 1986). Another line of research has characterized abnormalities in comprehending the high-level structure and meaning of sentences and discourse (for reviews, see Kuperberg, 2010b; Boudewyn et al., 2012). A largely separate literature has examined low-level sensory and perceptual deficits that affect the processing of the acoustic and visual features that make up spoken and written language (for a review, see Javitt and Freedman, 2015). A fourth line of research has focused on AVHs and the ability to distinguish one’s own inner speech from external speech (for reviews, see Ditman and Kuperberg, 2005; Moseley et al., 2013). These different dimensions of language research in schizophrenia have often been investigated by different researchers with different theoretical foci and investigative tools, and there have been few efforts to link them. Here, we articulate one possible approach for bringing these literatures together, based on an emerging *generative framework* for understanding typical language processing (see Kuperberg and Jaeger, 2015, for an overview).

Instead of focusing on how higher-order representations are assembled from lower-level building blocks, hierarchical generative frameworks of cognition focus on how higher-level inferences about the world constrain the interpretation of sensory information and how they are updated based on prediction error (e.g., Friston, 2005, 2010; Yuille and Kersten, 2006; Hinton, 2007; Clark, 2013). It has been proposed that a breakdown of generative circuitries is a fundamental abnormality underlying schizophrenia (Fletcher and Frith, 2009). Thus far, this theory has mainly been discussed in relation to the emergence of psychotic symptoms (e.g., Corlett et al., 2009, 2010; Fletcher and Frith, 2009). In this review, we explore the hypothesis that a breakdown of generative circuits proposed to underlie typical language comprehension and production can explain the multiple manifestations of language dysfunction in schizophrenia outlined above. We argue that this theoretical framework provides novel insights into potentially close relationships between abnormalities of multiple dimensions of language processing in schizophrenia. More generally, it highlights the importance of characterizing the links between abstract, high-level linguistic representations and low-level features of speech or text.

We begin by outlining generative models of cognition and their applications to understanding normal language comprehension and production. We then consider the generative circuit that links message level to lexical level representations, discussing how its breakdown might lead to impairments in comprehension in schizophrenia, and, in some patients, to thought-disordered language output. Next, we consider the generative circuits that further link message and lexical levels to low-level perception, focusing on how disrupted links between higher-level language representations and perceptual systems might lead to abnormal speech perception and to AVHs in schizophrenia. Finally, we dicsuss the implications of this generative framework for understanding neural abnormalities of language processing in schizophrenia, as well as its implications for real-world psychosocial functioning and cognitive remediation in schizophrenia.

## A Generative Framework of Language Comprehension and Production in Healthy Individuals

### Generative Frameworks of Cognition and Action: An Overview

At the core of generative frameworks are *internal generative circuits*. In its simplest form, a generative circuit functions to *infer* the underlying *cause* of a sensory signal (for reviews, see Knill and Pouget, 2004; Chater and Manning, 2006; Griffiths et al., 2010; for introductory overviews, see Jacobs and Kruschke, 2011; Perfors et al., 2011), and a dynamic generative circuit functions to incrementally infer the cause of a sensory signal that unfolds over time. At any particular moment in time, the agent may entertain multiple different hypotheses about this underlying cause and, to test these hypotheses, she can use them to actively generate *probabilistic predictions* about the sensorineural signal. At the next moment in time, the agent will observe more of the sensory signal, and any discrepancy between her predictions and the actual sensorineural signal — *prediction error* — can be used to *update her beliefs*, i.e., lead her to infer a new set of hypotheses about the underlying cause of the sensory signal. These hypotheses are, in turn, used to generate a new set of probabilistic predictions. By iteratively cycling between inference and prediction, with the aim of reducing prediction error, a dynamic generative circuit will converge upon the causal hypothesis that best *explains* the observed sensory signal (e.g., Friston, 2005; Hinton, 2007; Clark, 2013). While there are many different ways of formalizing prediction error, within a probabilistic computational framework, it is closely linked to belief updating and inference over the agent’s hypothesized perceptual and causal representations. Specifically, prediction error can be formalized as *Bayesian surprise* — the degree to which the agent’s belief *shifts* from her probabilistic predictions about the observed sensory signal (the prior) to her new probabilistic beliefs after observing new sensory input (the posterior), as determined by Bayes’ rule (Doya et al., 2007).

On its own, this type of very simple generative circuit cannot fully explain how we navigate our real-world environment. This is because, at any given moment, the sensory inputs that we encounter are enormously complex and multidimensional in nature. In order to explain such complex inputs, we can draw upon many different types of knowledge stored at multiple grains of representation. To give a concrete example, imagine conversing with a close friend, who says, “Let’s go out”. Your sensory input will include very complex patterns of acoustic and visual information that correspond to the particular features of your friend’s voice and face, information about sociolinguistic factors such as her age, gender, and ethnicity, as well as semantic and syntactic information about the content of the message itself. In order to infer the underlying cause of this complex pattern of sensory input (the fact that this particular friend has asked you to go out), you will need to draw upon multiple generative circuits, each corresponding to and linking these different types of representation, and organized in a hierarchical fashion at increasing levels of abstraction. Together, these linked generative circuits are said to comprise the agent’s internal *generative model* (Friston, 2005; Hinton, 2007; Clark, 2013). By linking perception with cognition, generative models can be used to home in on the underlying cause of complex patterns of sensory input by iteratively cycling between inference and prediction, as described above. Probabilistic predictions are actively propagated down the generative model at successively lower levels of representation, enabling more accurate (and often faster) recognition of consistent sensory input. Sensory information that hasn’t been accurately predicted — prediction error — is, in turn, passed up the model in a bottom-up fashion to successively higher levels of the generative model, and is used to update the agent’s beliefs about the underlying latent cause. This continues until prediction error across the entire generative model is minimized.

Importantly, we live in a world that is constantly changing and, because we do not have unbounded cognitive or metabolic resources or time, we must continually modify our goals and adapt to the broader situational context in which we find ourselves (see Qian et al., 2012 for discussion). One way in which an agent can rationally allocate her limited resources (see Simon, 1956; Howes et al., 2009; Griffiths et al., 2015) is to estimate the *precision* of her prior knowledge at any given level of representation, as well as the *reliability* of new inputs to that representational level (based on past experience), and use these estimates to modulate the *degree* to which she updates her beliefs (i.e., “weight” her prediction error, see Feldman and Friston, 2010; Friston, 2010). As we discus further below, this can, in turn, influence the general direction of information flow across the agent’s generative model (top-down versus bottom-up) and therefore the rate at which the agent *adapts* to her broader contextual environment.

Generative models not only link cognition and perception; they also link *action* and perception. This is because our knowledge of our own action plans can be used to generate predictions about intermediate action states as well as the sensory consequences of the planned action (cf. forward models, e.g., Jordan and Rumelhart, 1992; Guenther et al., 1994; Wolpert et al., 1995). Once again, the overarching goal of the agent is to home in on the representation at highest level of the hierarchy (the action plan) by minimizing prediction error (Bayesian surprise) across the entire generative model. And once again, the primary direction of information flow across the generative model can be influenced by the agent’s estimates of the precision of her priors and the reliability of new inputs (for discussion, see Adams et al., 2013a). As we will see later, such estimates also play an important role in allowing us to maintain the belief that we (rather than external events or agents) are the *source* of these action plans.

The hierarchical organization of generative models makes them particularly well suited as a theoretical approach to understanding the comprehension and production of language, which is composed of numerous hierarchically organized levels of representation. These representations include elemental sound units (*phonemes*), words (*lexical items*), the intonation and rhythm of phrases (*prosody*), the structure and meaning of sentences (*syntax, semantics*), and the relations between multiple sentences (*discourse*). These levels of representation essentially act as a code for transferring *message-level* representations from one person to another. The ultimate goal of language comprehension is to infer the message that the producer intended to convey from noisy and ambiguous sensory signals, and the ultimate goal of language production is to produce a sequence of such signals that efficiently and successfully *communicates* one’s intended message to other people’s minds. Below we discuss how the generative framework recasts the means by these goals are accomplished.

### A Generative Framework of Language Comprehension

Traditional frameworks have largely conceptualized language comprehension as a feed-forward *building* process: low-level features of the bottom-up perceptual input are mapped onto sublexical or lexical representations (e.g., Marslen-Wilson, 1987; Norris, 1994; Norris and McQueen, 2008; Grainger and Holcomb, 2009), which are, in turn, combined together to construct syntactic and semantic structures to build a higher message-level meaning (e.g., MacDonald et al., 1994; Jackendoff, 2007).

Within a generative framework, comprehension is conceptualized in quite a different way — as a process of generating top-down predictions in order to test hypotheses, and of updating these hypotheses based on new evidence (see Farmer et al., 2013, and Kuperberg and Jaeger, 2015, for discussion). Within this framework, our aim, first and foremost, is to *infer the latent cause* of incoming observations. We typically start out with high uncertainty about this cause. However, as new bottom-up evidence comes in, we update our higher-level hypotheses (beliefs) such that we home in on the cause of the inputs with increasing certainty (see, e.g., Levy, 2008, for a generative framework for syntactic parsing and Kleinschmidt and Jaeger, 2015, for a generative framework for phonetic adaptation). Within a hierarchical generative framework of comprehension (language understanding), the latent cause that the comprehender must infer is the underlying *message* that the speaker or writer intended to communicate, and the bottom-up input is the sensory signal, which can be encoded at multiple levels of representation (e.g., phonological, semantic, syntactic). Within an actively generative framework, we can test our higher-level hypotheses about the underlying message that causes this sensory signal by actively generating *predictions* at lower levels of representation, prior to the bottom-up input arriving and being decoded at these lower levels (see Kuperberg and Jaeger, 2015).^1^ For example, hypotheses about the overall message-level meaning can lead to predictions about upcoming words, which, in turn, can lead to predictions about upcoming acoustic-phonetic features. These predictions play an essential role in enabling us to extract signals from noisy channels in real time because they can “explain away” predictable aspects of the linguistic input. Any discrepancies between these predictions and the actual unfolding linguistic input — *prediction error* — are propagated back up the generative model, allowing us to incrementally *update* our higher-level hypotheses about the underlying message, enabling us to better infer the communicative intentions of the speaker or writer.

Importantly, our generative models are not fixed, but rather change constantly as we learn about and respond to our ever-changing environmental inputs. One way in which we may calibrate the rate at which we adapt language comprehension to the demands of any given communicative situation is to estimate the *precision* of our prior beliefs at a given representational level and the *reliability* of new inputs to that level (see Kleinschmidt and Jaeger, 2015, for related discussion). To give a concrete example, imagine meeting an unfamiliar person in a quiet room. In this situation, we expect our prior beliefs about that person’s voice to be relatively imprecise. However, based on our knowledge about sensory inputs in quiet rooms, we estimate the incoming sensory data to be fairly reliable. Based on these estimates, we should increase the degree of belief updating at perceptual levels of representation by relying more on new evidence than our prior beliefs. This means that there will be more prediction error (Bayesian surprise) being passed up to higher levels of the hierarchy, and so the flow of information across the generative model will primarily be *bottom-up*. This will allow us to learn from our environmental input relatively quickly, such that we converge rapidly on the underlying higher-level cognitive cause of the features of the speaker’s voice — associating these features with a specific speaker. This situation can be contrasted with conversing with an old friend at a noisy party. In this case, we estimate our prior beliefs about our friend’s voice to be very precise and, indeed, we may draw upon a generative model that corresponds directly to the features of our friend’s voice. On the other hand, based on our knowledge of noisy parties, we estimate any incoming sensory data to be unreliable. Together, these estimates can be used to ramp down belief updating — that is, we stick to our priors such that any prediction error is minimal. There will therefore be little passing of information up the generative model, and the flow of information across the generative model will be primarily *top-down.* This way of thinking about language highlights fundamental links between language processing, language acquisition and lifelong language adaptation (Chang et al., 2006; Jaeger and Snider, 2013; Dell and Chang, 2014).

### A Generative Framework of Language Production

Generative frameworks also conceptualize aspects of language production somewhat differently from classical frameworks. Traditionally, theories of language production have focused primarily on the processes by which messages are encoded at successively lower levels of representation, culminating in the articulation of linguistic signals (e.g., Dell, 1986; Levelt, 1993; Bock and Levelt, 1994). That is, the particular message that the producer plans is assumed to feed into production mechanisms that ultimately translate this message into auditory or visual signals. While there may be interactive activation between adjacent levels of representation during the encoding process, information primarily flows from higher to lower levels. Viewing the language production architecture instead in terms of hierarchically organized generative circuits places a stronger emphasis on *feedback* from lower to higher levels. Within this generative framework, just ahead of executing the actual speech plan, producers use their generative models to generate predictions at successively lower levels of representations (e.g., semantic, syntactic, phonological). Upon encountering new evidence, priors at each of these representational levels are updated to posteriors, and any shift in belief – Bayesian surprise or prediction error – is propagated back up the generative model, ensuring that the producer’s production plan is updated in real time. This feedback and updating plays a critical role in *monitoring* speech production (see also Pickering and Garrod, 2007, 2013).^2^ As we discuss below, the source of new evidence that leads to belief updating (and therefore prediction error) differs depending on whether monitoring is internal or external.

*Internal monitoring* ensures that any speech errors are edited out, and that the sounds, words and sentence structures that we actually produce are consistent with our original message-level intentions. It entails predicting our own production plans prior to articulation, and so the new incoming evidence that leads to updating of prior predictions comes from the execution of the speech plan itself. For example, predictions (priors) at the phonological level will be updated when the production plan is encoded at this level, and any resulting shift in belief (prediction error) will be passed up the model, leading the speaker to rapidly update her beliefs at higher levels of representation “on the fly” – either the message itself, or her semantic or syntactic representations. As a result, the message the speaker conveys, or the semantic/syntactic representations used to express this message, will be congruent with the phonemes that she has selected for articulation (for evidence for adaptive internal monitoring, see Severens et al., 2011; Dhooge and Hartsuiker, 2012), and prediction error will be reduced across the entire generative model. Moreover, in addition to this type of internal feedback monitoring, speakers also have at their disposal another way of reducing prediction error across the generative model: they can actually *act* on their environment to fulfill their own predictions, resulting in *production facilitation*. For example, predictions at the phonemic level may be used to facilitate the selection of phonemic information encoded by the speech plan itself, ensuring that the precise elements that are ultimately produced are maximally compatible with the speaker’s original message-level intentions^3^.

Important factors that may influence the route by which prediction error is minimized – internal feedback monitoring or production facilitation – are the producer’s estimates of the *precision* of her prior beliefs (e.g., determined by the specificity of her communicative goals) and her estimates of the *reliability* of new evidence (e.g., determined by demands on cognitive resources or her past experience with her own productions). As discussed above for comprehension, these estimates of precision and reliability are thought to influence the degree of belief updating at any given level of representation, effectively weighting the prediction error. Internal feedback monitoring is most likely to occur if a speaker has estimated her production plan to be relatively imprecise (e.g., because her communicative goals are relatively general). In this case, shifts in belief (prediction error) will be large and will be propagated up the generative model, leading the speaker to adjust her speech plan in real time, as described above. In contrast, production facilitation is most likely to occur if the speaker has estimated her production plan to be highly precise (e.g., because her message-level intention is very specific). In this case, the flow of activity across the producer’s generative model will be primarily top-down, with any small remaining prediction error largely resolved by using predictions to facilitate the selection of information at relatively low levels of representation.

*External monitoring* ensures that our speech output is calibrated to the needs of the comprehender. It entails predicting the perceptual consequences of the output that we produce, e.g., the sounds of our own voices, as well as our interlocutor’s comprehension, and so the new incoming evidence that leads to updating of prior predictions comes from the external world, just as in comprehension. So, for example, if we had predicted that our voice would sound loud, but the auditory feedback we receive indicates that it sounds soft, then a relatively large prediction error will be passed up the generative model leading us to speak louder. Or if we had estimated that our comprehender would understand what we had said, but the feedback we receive suggests otherwise, then the resulting large prediction error will pass up the generative model, leading us to update our speech plan (either the message itself, or the semantic or syntactic representations we use to express it), thereby enabling us to adapt our production to our interlocutors’ abilities in real time. In this way, we can improve the odds of successful communication.

Although prediction errors play a critical role in language production by enabling monitoring, it is important to note that these errors will generally be small, and much smaller than the prediction errors produced during comprehension. This is because when we produce our own speech, we have access to our own communicative intentions, our utterance plans, and even our articulatory motor plans, and so our predictions will generally be highly accurate, “explaining away” much of the effects of our own speech plans and the consequences of our articulation. Moreover, we will generally estimate our predictions to be more reliable during our own productions than during comprehension when we lack direct access to our interlocutor’s message-level intentions and speech plan. This means that, even if the prior and input are kept constant at any given level of representation, prediction errors that result from self-generated linguistic input will generally be smaller (and therefore down-weighted) than prediction errors that result from externally generated linguistic input. At a perceptual level, this attenuation of prediction error can be detected experimentally as reduced neural response evoked by sensory inputs resulting from self-generated speech versus other-generated speech (e.g., Creutzfeldt et al., 1989).

The attenuation of prediction error during production versus comprehension is thought to play an important role in helping us maintain the inference that our own speech production originates from ourselves rather than from external agents (for discussion in relation to self-monitoring of action more generally, see Blakemore et al., 1999; Martikainen et al., 2005; Bays et al., 2006; Aliu et al., 2009; Cardoso-Leite et al., 2010; Hesse et al., 2010). As we will discuss further below, a breakdown in this self-monitoring may lead to the phenomenon of AVHs in schizophrenia.

## Higher Order Language Dysfunction in Schizophrenia: Disruptions in the Generative Circuit Linking Message-Level and Lexical Representations

In this section, we propose that a breakdown of the generative circuit that links message-level to semantic and lexical representations contributes to the phenomenology of thought disorder, as well as to analogous abnormalities of higher-order comprehension in schizophrenia.

Positive thought disorder — the incoherent and disorganized language output that is seen in some patients — is perhaps the most obvious manifestation of language dysfunction in schizophrenia. Thought disorder manifests variably across patients (and sometimes within an individual patient across different communicative situations). It can include tangential leaps in the message being expressed (Bleuler, 1950; Chaika, 1974; Andreasen, 1979a,b, 1986) as well as a so-called “loosening of associations” (Bleuler, 1950) – a tendency to produce semantically associated words that are only indirectly related or completely unrelated to the overall message being conveyed (Andreasen, 1979a,b, 1986).

Although thought disorder manifests clinically in only a subset of schizophrenia patients, there is now a body of work suggesting that patients, both with and without clinical evidence of thought disorder, can show a set of related phenomena during language *comprehension* including a relative insensitivity to overall sentence (Kuperberg et al., 1998, 2006a) and discourse (Ditman et al., 2011) coherence, and an over-dependence on the individual meanings or semantic relationships between individual words (for reviews, see Kuperberg, 2010a,b; Boudewyn et al., 2012). Here, we first discuss how such higher-order comprehension abnormalities can be understood as arising from a disruption of the generative circuit that links message-level and lexical representations. We then relate this perspective to the production phenomena associated with thought disorder itself.

### Abnormalities of Higher-level Language Comprehension in Schizophrenia

In healthy individuals, a generative circuit between message-level and lexical representations plays a crucial role in real-time language comprehension. As language unfolds word by word, we use our message-level hypotheses (based on the preceding linguistic and non-linguistic context and our real world knowledge) to generate predictions about the most likely semantic features of upcoming words, and the meanings of incoming words are assessed in the light of these predictions. This fast and pro-active use of context allows us to rapidly resolve the inherent *ambiguity* of incoming words. For example, when processing lexically ambiguous *homophones* (words that share the same sounds but differ in meaning, e.g., “bank”) in isolation, we normally activate both the dominant meaning (“financial institution”) as well as less frequent subordinate meanings (e.g., “terrain alongside a river”). We also activate its *semantic neighbors* — other words that are semantically associated with and/or share semantic features with each of these different senses of “bank”. However, encountering “bank” in a sentence context that biases toward its subordinate meaning (“river bank”) will lead to this subordinate meaning (and its associated semantic neighbors) being activated at the very earliest moments of semantic processing (Li and Yip, 1998; Huettig and Altmann, 2007; Calacouris and Brock, 2009)^4^.

We have known since classic experiments by Chapman et al. (1964) that patients with schizophrenia are impaired in their ability to correctly interpret the contextually appropriate meaning of homophones. More recent studies have established that such impairments manifest very quickly during real-time comprehension (e.g., Titone et al., 2000; Sitnikova et al., 2002). For example, when an ambiguous word appears in a context that biases strongly toward its subordinate interpretation, healthy adults show electrophysiological signatures of rapid contextual facilitation of subsequent words that are semantically congruent with this meaning. In contrast, patients’ neural responses are primarily determined by the more dominant meaning rather than the contextually supported meaning (Sitnikova et al., 2002). This finding is consistent with the idea that, unlike healthy controls, patients fail to use message-level representations to predict the subordinate features of the homographs.

A breakdown in this generative circuitry may also help explain the abnormal activation of semantic neighbors in schizophrenia. As noted above, the context in which we encounter any given word will constrain which of its semantic neighbors we activate and to what degree. If patients fail to use generative circuits to predict an upcoming semantic input, they might indiscriminately activate a much broader set of semantic neighbors when new input is encountered. This may lead them to over-rely on semantic associations between individual words to make sense of language during word-by-word comprehension.^5^ On this account, patients’ impairments in their use of higher-order context should be *functionally linked* to their over-dependence on semantic associations (Kuperberg et al., 2006a; Ditman et al., 2011; Swaab et al., 2013). Consistent with this idea, an over-reliance on lexical associations can lead patients to come to erroneous interpretations of sentences (Kuperberg et al., 2006b) or to establish inappropriate referential links across sentences (Ditman et al., 2011).

Finally, a breakdown in this generative circuitry may help explain patients’ difficulties with comprehending non-literal language. This is because, in healthy individuals, this circuitry may aid our comprehension of certain types of figures of speech. For example, when processing the sentence “John had a long happy life, but last week he finally kicked the bucket”, we normally use our message-level hypotheses to predict the idiomatic expression, “kicked the bucket”, as a whole, without needing to first activate each incoming word from the bottom-up to construct its literal meaning. Moreover, in comprehending less conventionalized non-literal language, such as unfamiliar metaphor, we may benefit from switching to or learning new generative models that allow us to infer the intended underlying meaning. In schizophrenia, a breakdown of generative mechanisms may account for a tendency to interpret metaphoric or figurative language in overly literal ways (Chapman, 1960; Titone et al., 2002; Brune and Bodenstein, 2005; Kiang et al., 2007).

Importantly, a breakdown in the generative mechanisms that normally bridge message-level hypotheses and lexical activation does not imply that patients cannot use higher-order context at all. The crucial point is that in healthy individuals, generative circuits act very rapidly by generating predictions *before* new input is fully accessed from the bottom-up. While patients may be unable to mobilize context quickly enough to generate such predictions, they may still be able to use a higher message-level representation to influence interpretation *after* the bottom-up input has been fully accessed. Some preliminary evidence for this hypothesis comes from a study in our lab investigating the interpretation of ambiguous spoken instructions (Rabagliati et al., 2014). We showed that patients with schizophrenia were able to effectively use two different types of high-level context (conversational discourse context and broad visual context) to influence their final interpretation of these spoken instructions. However, examination of measures that reflected word-by-word comprehension revealed that, unlike healthy adults, patients failed to use these high-level contexts to influence their incremental interpretation of the instruction as it unfolded in real time.

Exactly why schizophrenia patients seem unable to use strong prior beliefs to predictively pre-activate upcoming input as quickly as healthy individuals remains unclear. One hypothesis, however, is that the impairment stems from a general tendency of patients to underestimate the *precision* of their prior predictions (see Adams et al., 2013b). As discussed above, the ability to use such estimations of precision may play an important role in modulating the *degree* of Bayesian updating for a given prior and likelihood function (weighting the prediction error). This, in turn, ensures that the balance of top-down versus bottom-up information flow across a generative model is calibrated to our broader contextual environment. In schizophrenia, a tendency to discount the precision of prior predictions would lead to an overweighting of prediction error and to a relative over-dependence on bottom-up activity. Thus, patients would engage with language in a primarily *reactive* fashion, rather than in a more efficient predictive fashion.

Many of the comprehension abnormalities described above are seen in patients both with and without clinical manifestations of thought disorder. There is, however, some evidence for processing abnormalities that are specific to thought disorder: under conditions that encourage automatic stimulus processing, thought disordered patients can show larger automatic semantic priming effects than non-thought disordered patients and healthy controls, particularly when the semantic associations in question are *indirect* – that is, when the prime and target words are not themselves related, but are both semantically related to a non-presented mediator word (e.g., *lion* – *stripes*, where the mediating word is *tiger*; Spitzer et al., 1993; Weisbrod et al., 1998; Moritz et al., 2001, 2002; Kreher et al., 2009).^6^ This has been taken as evidence that thought disordered patients exhibit more widespread activation of semantic networks to words presented in isolation (the prime words in these studies) than healthy adults or patients without thought disorder.

The finding that even words presented in isolation can evoke abnormal patterns of semantic activation in thought disordered patients may initially seem somewhat at odds with the generative perspective, which we have thus far discussed in relation to patients’ difficulties with interpreting language in context. However, they make more sense if we consider the clinical manifestation of thought disorder as reflecting a particularly severe breakdown of generative circuits. As noted in the previous section, generative circuits function not only to ensure efficiency of language processing; they also play a critical role in allowing us to learn and *adapt* our internal representations. As discussed above, disruptions of generative models in schizophrenia would lead to highly abnormal and unresolved lexical prediction errors to words presented in context. If this disruption was particularly severe, then, over time, these abnormal prediction errors might interfere with the maintenance of stable lexical representations. Thus, rather than being finely tuned for mapping specific lexical forms on to specific semantic features, lexical mappings would become noisier. Therefore, a particular lexical input, even when presented in isolation, might inappropriately activate multiple related semantic representations, leading to indiscriminate and unconstrained semantic activity. This would manifest as an overly inclusive lexico-semantic network, which is consistent with the observations described above (see also Mathalon et al., 2002, 2010).

As we will discuss next, this type of severe breakdown of generative circuits linking high-level message representations and lexical representations might also impact language *production* in schizophrenia, thereby leading to the clinical manifestation of thought disorder itself.

### Abnormal Language Production: Toward an Understanding of Thought Disorder

Within a generative framework, the essential goal of language production is to communicate an intended message to one’s interlocutor’s mind with minimum error during transmission (Jaeger and Ferreira, 2013). To do this successfully, the speaker generates predictions about the internal and external *consequences* of her message plan at multiple levels of representation, and she can use this prediction error to adjust the language production process itself (internal and external monitoring).

Within this framework, the generative circuit linking message-level and lexical representations plays a critical role in ensuring that the words selected during production are congruous with the message that the speaker aims to convey. If the speaker is not quite certain of what she intends to say and has therefore estimated her message-level predictions to be relatively imprecise, then any discrepancies between her predictions and activity at the lexical level when the production plan is actually encoded (prediction error signals) will be propagated back up the generative model and used to adjust her message and production plan such that they are consistent with the lexical choice that she has selected (internal monitoring). If, however, the speaker has estimated that her intended message and its downstream encoding are highly precise, then any top-down predictions based on this message may actually be used to facilitate the selection of those lexical items that are most consistent with the overall message, thereby ensuring that inappropriate lexical associates are not articulated. Together, both these routes ensure that the overall prediction error across the generative model is minimized, that free-associative lexical activity is excluded, and that the message communicated is coherent with respect to the speaker’s intended message (see also Dell, 1986; Dell and Chang, 2014).

In schizophrenia, a reduction in the propagation of top-down predictions down the generative model, and an over-dependence on bottom-up processing, would therefore lead to the following consequences. First, the resulting inappropriately large prediction error would feed back up to the message-level representation, triggering inappropriate shifts in the message-level representation — an over-adjustment of the production plan. This would lead to the tangentiality and derailment that characterize thought-disordered speech. Second, lexico-semantic selection would be relatively unconstrained, with lexical associates that are incongruous with the overall message going largely uncorrected and intruding into speech output. This would lead to the ‘loosening of associations’ that can also characterize thought disorder. As discussed above, it is possible this reduced prediction in patients stems from a general tendency to underestimate the *precision* of their own speech plans (see Adams et al., 2013b, for simulations of a simple communication system using a predictive coding formalization, which provide evidence that a reduction of precision at higher levels of a generative model can reduce the capacity to make accurate lower-level predictions, rendering all new inputs surprising).

Finally, although most work has focused on the generative circuit linking message-level and lexico-semantic representations, it is possible that a disruption of other generative circuits during language production might also contribute to the phenomenology of thought disorder. For example, a disruption of the circuitry linking message or semantic-level representations to phonological representations may sometimes lead to the intrusion of phonologically related items into the speech output (so-called “clang associations”; Bleuler, 1950; Chaika, 1974; Andreasen, 1979a; Spitzer et al., 1994). And a failure to monitor signs that the comprehender is not grasping the intended message might contribute to the failure of *communication* that can characterize thought disorder (Chaika, 1974; Harrow et al., 1989). Indeed, if, as has been hypothesized, the prediction error that generative models in production aim to minimize is the outcome of the entire inference process that constitutes comprehension (Jaeger and Ferreira, 2013; Lind et al., 2014a), then a failure to take the comprehender into account may, to a large extent, drive abnormal monitoring at other levels of the system in thought disorder (for early discussions of this idea, see Cohen, 1976; Harrow et al., 1989; MacGrath, 1991).

## Speech Perceptions and Auditory Verbal Hallucinations: Disruptions in the Generative Circuit Linking Higher-Level Linguistic Representations and Low-Level Perception

In this section, we extend the proposal introduced in the previous section by suggesting that a disruption of the generative circuits that link message-level and lexical representations extends all the way down to lower-level perceptual representations, and that this might help explain both the phenomena associated with AVHs as well as the perceptual language abnormalities observed in schizophrenia.

The phenomenon of hearing voices — the perception of speech in the absence of an actual external speech stimulus — is the most common symptom of schizophrenia overall, affecting an estimated 60–70% of patients (World Health Organization, 1973; Andreasen and Flaum, 1991). It is important to recognize that AVHs are not merely arbitrary sounds; they are fully linguistic in nature, often containing rich detail at multiple levels of linguistic representation, ranging from specific, elaborate message-level content to auditory perceptual and prosodic features that distinguish different “speakers” along dimensions such as gender, age, accent, and speaking style (Nayani and David, 1996; Garrett and Silva, 2003; Stephane et al., 2003).

While AVHs are the most obvious clinical manifestation of perceptual language disturbances in schizophrenia, there is also evidence that patients, even those without AVHs, experience low-level sensory and perceptual disturbances (e.g., Adler et al., 1982; Holcomb et al., 1995; Rabinowicz et al., 2000; Turetsky et al., 2009; Micoulaud-Franchi et al., 2011) including abnormalities in the perception of speech sounds.^7^ These speech perception abnormalities have mostly been discussed and investigated separately from the higher-order language comprehension abnormalities discussed in the previous section. They have also been considered separately from the literature on AVHs. Here, we discuss the possibility that these lower-level processing abnormalities can be understood as arising from a disruption of the generative circuits that link message-level and lexical representations all the way down to low-level perceptual representations. We then relate this perspective to the phenomenon of AVH itself.

### Abnormalities of Low-level Speech Perception in Schizophrenia

Speech is an acoustically complex and rapidly time-varying auditory signal whose basic elements – phonemes – differ from each other along numerous but very subtle acoustic dimensions (e.g., Ladefoged, 1975). It is therefore unsurprising that schizophrenia patients’ difficulties with perceiving and categorizing low-level auditory stimuli (see Introduction) extend to these complex speech sounds. Patients have less precise representations of how acoustic features map onto phonemes than healthy adults (Cienfuegos et al., 1999), and also show impairments in mapping these features onto more complex or abstract representations, such as emotional tone of voice (Leitman et al., 2005).

As noted above, low-level auditory and speech perception deficits in schizophrenia have generally been investigated independently of higher-level language processing abnormalities. To the extent that some researchers have considered potential relations between speech perception and higher-level language processing, they have hypothesized that early perceptual and sensory abnormalities in schizophrenia are the root cause of upstream disruptions to such higher-order representations and processes (e.g., Javitt, 2009; Javitt and Freedman, 2015). The evidence taken to support this hypothesis comes from observations that patients’ ability to perceive emotional or sarcastic tones of voice is correlated with their low-level pitch perception and duration discrimination abilities (Leitman et al., 2005; Jahshan et al., 2013; Kantrowitz et al., 2014). Similarly, reading dysfunction in schizophrenia is correlated with low-level auditory and visual perception abilities (Revheim et al., 2014). However, these observations have generally been limited to correlational effects in a handful of domains, leaving open questions about the scope and causal direction of relations between perception and interpretation abnormalities. In fact, the observed phenomena are just as consistent with the converse view: that disrupted generative models are the primary deficit, with low-level perceptual language abnormalities in schizophrenia stemming from a failure of generative models to accurately *predict* incoming sensory data (see Hemsley, 1993, for similar ideas).

In healthy individuals, there is now a large body of evidence suggesting that the accurate perception of speech sounds is highly dependent on a dynamic use of contextual information (e.g., Newman et al., 2001; Allen et al., 2004; Pardo and Remez, 2006; McMurray and Jongman, 2011). This is necessary for us to overcome a major computational challenge to spoken language comprehension — the so-called *lack of invariance problem* (Liberman et al., 1967). The /b/ in “ba”, for example, has quite different acoustic features from the /b/ in “bee” (Delattre et al., 1952). And two different speakers saying “ba” may (and probably will) produce considerably different acoustic patterns (e.g., Johnson and Wright, 1990), to the extent that one speaker’s “ba” might effectively be another speaker’s “pa”. Yet, despite this pervasive acoustic variability and ambiguity, we are remarkably good at interpreting acoustic patterns as the phonemes that the speaker intended to say.

Generative circuits linking message-level and perceptual representations can help us solve this lack of invariance problem by enabling us to predict not only the meanings of words (as discussed in the previous section), but also the *sounds* of upcoming information in the speech signal. These perceptual predictions constrain our interpretations of incoming acoustic information and can even “fill in” gaps in the speech stream (Warren, 1970). To generate such perceptual predictions, we are able to draw upon any representation that is higher than the lowest level perceptual representations. For example, we can use *lexical* knowledge to make perceptual inferences about ambiguous speech sounds: a speech sound that is acoustically ambiguous between /f/ and /sh/ is generally perceived as /f/ when it is presented in a string like *da?odil* and as /sh/ when it is presented in a string like *na?ional* (Ganong, 1980). We can also use *message-level* representations to predict and infer the phonology of particular words (DeLong et al., 2005). And we routinely use our knowledge about *individual speakers* and *groups of speakers* to make more general predictions about pitch contours, rhythm, and speech style, even when we do not have strong predictions about the exact phonemes we will hear (e.g., King and Sumner, 2014; Brown et al., 2015a,b; for a recent review, see Kleinschmidt and Jaeger, 2015).

A second way in which these generative circuits can resolve the lack of invariance problem is by enabling listeners to quickly *adapt* to their current linguistic environment. As discussed in Section “A generative framework of language comprehension and production in healthy individuals,” any errors in our predictions will lead not only to more effective inference, but also to learning and adaptation either by *adjusting* our knowledge about the contingencies or mappings between the representations that define our existing generative models, or by *switching to* a different generative model that better describes the statistical properties of the input. At the phonemic level, for example, encountering many slightly mispronounced versions of a phoneme (e.g., /b/) will lead to systematic prediction errors at the phonemic level. These will, in turn, lead listeners to adjust their internal mappings from cue to category to accommodate future deviant pronunciations (*phoneme recalibration*; Norris et al., 2003; Kraljic and Samuel, 2005). The converse is also true: hearing several highly prototypical pronunciations of /b/, without the natural variation in production that listeners typically encounter, leads listeners to narrow their /b/ representation to exclude even slightly deviant pronunciations (*selective adaptation*; Vroomen et al., 2004; see Kleinschmidt and Jaeger, 2015 for a computational model).

In schizophrenia, there is little work examining effects of context on speech perception. However, an early study suggested that patients’ speech percepts deviate much more strikingly from those of healthy adults when words are presented in a connected narrative than when they are presented one at a time (Lawson et al., 1964). Moreover, speech processing deficits in schizophrenia are especially pronounced when the task involves perceiving speech in noisy conditions (Hoffman et al., 1999; Wu et al., 2012) – conditions in which healthy adults rely particularly strongly on contextual prediction (e.g., Davis and Johnsrude, 2007).

There is even less consensus concerning patients’ ability to generate predictions or use prediction error to adjust their phoneme representations. On the one hand, when several /b/s are followed by a /p/, patients with schizophrenia show a smaller mismatch negativity effect than that of control participants (Kasai et al., 2002), suggesting abnormal perceptual error signals to unexpected phonemes (see Todd et al., 2013 for a more general review of the mismatch negativity in schizophrenia). On the other hand, patients show apparently normal selective adaptation effects when exposed to phonemes in isolation (see Cienfuegos et al., 1999, although it is possible that the parameters of the paradigm used in this study indexed local contrast effects rather than true phoneme adaptation; see Samuel, 1986, for evidence that such mechanisms can drive effects in this paradigm under certain conditions).

If, as we hypothesize, low-level perceptual abnormalities in schizophrenia are related to a dysfunction of generative circuits, this raises numerous questions about what types of higher-level dimensions of language comprehension (such as syntax, semantics, pragmatics, and discourse) patients are able to take into account when processing low-level speech sounds (as well as low-level visual representations). Given that we rarely hear isolated speech sounds in everyday communication, addressing these questions would likely provide additional insight into patients’ capacities in a wider range of real-world communicative contexts. Paradoxically, a reduced use of higher-level representations to interpret speech sounds could lead to situations in which patients with schizophrenia might perceive speech more veridically than healthy adults. For example, it may be the case that patients are in fact “better” at perceiving prototypical pronunciations of phonemes (i.e., how they would be pronounced in isolation) in contexts in which typical participants would expect non-prototypical pronunciations (e.g., due to phonetic overlap with surrounding phonemes, or due to speaker-specific factors such as foreign accent).

Finally, it is possible that a particularly severe breakdown of these generative circuitries between higher-level and low-level perceptual representations might, over time, lead to weaker or noisier mappings between perceptual inputs and the low-level phonetic or phonemic representations themselves. In other words, if patients with schizophrenia are less able to leverage sentence and word context when processing speech stimuli, then this would lead to a failure to appropriately *adapt* not only at the level of these higher-level representations, but also at levels lower down the hierarchy. Inappropriately large prediction error to predictable phonemes presented in context would mean that, over time, rather than maintaining fine-grained mappings between acoustic inputs and phonemic and phonetic representations, these mappings would become noisier and inappropriately activated, even to phonemes and words presented in isolation. Thus, even though the generative perspective predicts that patients would show *more* difficulties with perceiving speech sounds in context, some patients may also show a degradation of low-level perceptual representations, with changes in their sensitivity to sensory stimuli even when presented in isolation. This would imply that disruptions in aspects of low-level speech might arise as a downstream consequence of disruptions in higher-order generative models. And, if this is the case, auditory and speech processing deficits would be expected to correlate with higher-level comprehension abilities in schizophrenia, as has been reported (Leitman et al., 2005; Jahshan et al., 2013; Kantrowitz et al., 2014; Revheim et al., 2014).

### Where Perception Meets Production: Toward an Understanding of Auditory Verbal Hallucinations

We now turn to the question of how a severe breakdown of the generative circuits between high-level message representations and low-level perceptual representations might impact aspects of language *production* in schizophrenia, and account for the clinical manifestation of AVHs.

The generative framework of language production that we outlined above entails monitoring (via prediction and updating) of both production-internal mechanisms as well as the perceptual consequences of our own speech plans in order to ensure that the intended message reaches the interlocutor’s mind with minimum error during transmission (Jaeger and Ferreira, 2013). This whole process may well draw on the same generative circuits discussed above that link message-level and lexical representations all the way down to lower-level phonological and perceptual representations.

As discussed above, our prediction errors are much smaller when we hear ourselves speaking than when we hear others speaking. This is both because our predictions about the effects of our own speech plans are much more accurate than our predictions about the effects of others’ speech, and because we estimate our own speech plans to be highly reliable, leading us to attenuate any belief updating (and down-weight prediction error). This attenuation of prediction error to self-generated speech is thought to play an important role in allowing us to maintain a strong sense of *agency* over our own speech production — both the speech we produce (Sugimori et al., 2013; Lind et al., 2014a), as well as the phonological representations and articulatory representations that may be activated without actual speech production (i.e., covert speech)^8^.

In schizophrenia, a severe breakdown of the generative connections between message-level, semantic, phonological, and perceptual representations may disrupt the causal links between the intention to speak (whether overtly or covertly) and the predicted consequences of these speech plans, resulting in relatively large prediction errors at multiple levels of the generative hierarchy. That is, patients may be less able to use stored higher-level representations to predict the consequences of activating semantic, syntactic phonological and articulatory representations ahead of encoding the speech plan at these levels, possibly because they underestimate the precision of higher-level message representations, as discussed above (see Adams et al., 2013b). Further, patients may also under-estimate the reliability of activity at the lower perceptual levels, possibly as a secondary consequence of reductions in estimates of precision at higher levels of the hierarchy (see Adams et al., 2013b, for evidence using simulations of simple communication systems). These reduced estimates of reliability of activity at low-level perceptual representations would lead to relatively more belief updating at these levels (an up-weighting of prediction error) and an increased bottom-up flow of activity up the generative hierarchy to self-generated messages, just as in comprehension. This, in turn, may alter patients’ inferences about the *source* of the entire production, such that they infer that it originates from some unknown external agent, rather than from themselves. From this perspective, AVHs may be abnormal percepts that result from failures to explain away both the sensory and linguistic consequences of self-generated messages (see also Fletcher and Frith, 2009; Adams et al., 2013b).

Some evidence to support this idea comes from studies of perceptual external self-monitoring during speech production in schizophrenia. First, in series of ERP studies, Ford and Mathalon (2005) and Ford et al. (2007) compared a sensory ERP waveform (the N1 component) to self- vs. externally generated speech sounds. The N1 ERP component elicited by the onset of a speech sound is normally attenuated when the speech sound is produced by the participant, rather than by another speaker, reflecting the attenuation of a sensory prediction error. This was not the case in schizophrenia, supporting the idea that patients may fail to attenuate sensory prediction error resulting from self-produced speech. Further, this lack of attenuation was associated with reduced neural synchrony between frontal and temporal brain regions, suggesting a neural dissociation between generative models and low-level perceptual cortices (Ford et al., 2007). Of note, both these abnormalities were observed not only in patients experiencing AVHs, but also in non-hallucinating patients (Ford and Mathalon, 2005).

Second, another series of studies examined patients’ ability to identify the source of self-generated words after artificially inducing a large perceptual prediction error. This was done by routing participants’ vocal output through a device that distorted their speech and playing it back to them over headphones in real time, so that no delay was detected between speaking and hearing (e.g., Johns and McGuire, 1999; Johns et al., 2001; Allen et al., 2004). The main finding from these studies was that patients were more likely to misattribute their own distorted speech to an external voice than healthy controls, particularly when the words in question were derogatory (as opposed to neutral or positively valenced; Johns and McGuire, 1999; Johns et al., 2001). Unlike the studies by Ford and Mathalon (2005) and Ford et al. (2007), this abnormality appeared to be fairly specific for patients experiencing AVHs. This may be because participants were producing words with semantic content, which is more likely to have engaged the circuitry that links semantic to perceptual representations — a component of the generative circuitry that we hypothesize to be disrupted in patients with AVHs.

Importantly, the theory advanced here goes beyond the idea that AVHs arise from a specific breakdown of a *perceptual* self-monitor that links intentions to generate inner speech with percepts (Frith, 1992). Rather, by conceptualizing AVHs in schizophrenia as originating from a breakdown in hierarchically organized generative circuits, it predicts that patients with AVHs will show abnormally large prediction errors not only at the level of percepts but also at higher levels of the generative hierarchy. Some preliminary evidence consistent with this idea comes from observations that patients with schizophrenia use fewer first-person pronouns and self-oriented content words, and more third-person pronouns and externally oriented content words, than patients with mood or anxiety disorders (Fineberg et al., 2015a). It is possible that, as patients become more likely to attribute their own speech to an external source, the content of their utterances likewise develops an increasingly external focus.

It will, however, be important for future studies to directly test the hypothesis that patients with AVHs show abnormalities of monitoring at higher levels of representation. One fruitful avenue of research may be to take advantage of a novel form of real-time speech perturbation that has been used to examine how high-level semantic monitoring is affected by auditory feedback from one’s own voice (Lind et al., 2014a,b). In these experiments, rather than merely perturbing the acoustic features of a participant’s output during articulation, auditory feedback from the participant’s voice is replaced entirely by a recording of his own voice uttering an entirely different word (e.g., the speaker says “gray” and hears “green”). Many healthy participants actually fail to detect these incongruences, remaining blind to the real-time speech manipulation. Further, when probed to repeat their response, on many non-detected incongruent trials, they explicitly report having said the substituted word, even though their initial production was in fact a different word. In other words, on these trials, healthy participants appear to have updated their own initial communicative intentions on the basis of the perceptual prediction error, to maintain the inference that they were the source of the word they heard. This supports the idea that there are close inferential links between speech perception and semantic self-monitoring in healthy adults, as discussed above. If, as we hypothesize, a breakdown in the circuitry linking message-level and perceptual representations contributes to AVHs in schizophrenia, then, despite perceptual prediction error being larger than in controls, patients may fail to update their own message-level production plans on the basis of this prediction error, and, as a result, may be *less* likely to report having said the substituted word on these trials. That is, they may be more accurate than control participants in their reports of what they actually said^9^.

Finally, patients who have difficulties with determining the source of their own speech might also have difficulties with perceiving and distinctly representing speech from multiple (external) talkers. That is, if AVHs arise from abnormalities in the generative architecture that normally enables us to determine the source of linguistic signals, source attribution problems should not be limited to the ability to discriminate between one’s own speech and external speech. Instead, breakdowns in generative circuits supporting inferences about the sources of linguistic signals should lead to more general problems with using these mechanisms to discriminate between multiple potential speakers. This would further compound the difficulties that patients experience in real-world communicative situations.

## Implications for Understanding the Neurobiological Underpinnings of Language Dysfunction in Schizophrenia

Framing language dysfunction in schizophrenia in terms of generative models connects with neurobiological evidence that schizophrenia is a disconnection syndrome, characterized by abnormalities in both structural and functional brain connectivity (Friston and Frith, 1995; Friston, 1998; Stephan et al., 2006; Cannon, 2015). In healthy individuals, predictive processing of the unfolding linguistic input and the use of prediction error to update message-level representations are? thought to depend crucially on the integrity of fast, parallel connections between perceptual cortices and the neural loci of stored language knowledge at multiple hierarchical levels of representation, across prefrontal, temporal and parietal cortices (Hickok and Poeppel, 2007; Kuperberg, 2007; Baggio and Hagoort, 2011; Friederici, 2012; Price, 2012; Hagoort, 2013). Patients with schizophrenia exhibit structural changes across the prefrontal, temporal, and inferior parietal cortices that make up this language network (Shenton et al., 2001; Kuperberg et al., 2003; Wisco et al., 2007; Catani et al., 2011; Schneiderman et al., 2011; Abdul-Rahman et al., 2012). Patients also show abnormal functional connectivity between these regions during the resting state (e.g., Garrity et al., 2007; Hinkley et al., 2010; Liemburg et al., 2012). A full understanding of how such functional dysconnectivity gives rise to the symptoms and specific perceptual and cognitive dysfunctions that characterize schizophrenia requires the use of paradigms that specifically probe the relevant cognitive processes. While a full discussion of this work is beyond the scope of this review, we highlight a few lines of research that may be particularly relevant to generative circuitries discussed in the sections above.

### The Generative Circuit Linking Message-level and Lexical Representations

First, consider the message-to-lexical generative circuit. In healthy adults, different aspects of lexical processing may be mediated by different ‘hubs’ within the temporal cortex. For example, the left posterior middle/superior temporal cortex (post-M/STG) may play a role in mapping the word-form (e.g., phonological) on to syntactic and semantic representations (Hickok and Poeppel, 2007; Martin, 2007; Lau et al., 2008; Binder et al., 2009); the left anterior superior temporal cortex may map word-form on to semantic representations; and more inferior parts of the anterior temporal cortex may play a role in mapping widely distributed conceptual-semantic features on to amodal semantic representations (Patterson et al., 2007; Price, 2012; see also McCarthy et al., 1995; Nobre and McCarthy, 1995). All these temporal regions are reciprocally connected to frontal cortices. For example, the left post-M/STG shows both functional and structural connections with the left inferior frontal cortex (Catani et al., 2007; Snijders et al., 2010), and these two regions are often co-activated during lexico-semantic processing (e.g., Rodd et al., 2005, 2012; Gold et al., 2006). Integrating what we know about this frontotemporal language processing network with our assumptions about how generative models function in the brain, we speculate that, during higher-level comprehension, activity evoked within temporal regions by incoming words is *attenuated* to be commensurate with how accurately and confidently we have already used to context to predict the semantic, syntactic and word-form properties of the incoming word. For example, large prediction error signals at the level of syntactic, semantic and word-form features (e.g., to an incoming word following a sentential context that does not constrain for any of these properties) may be associated with increased activity within the left posterior M/STG. In contrast, large prediction error signals at the level of just semantic and word-form features (e.g., to incoming words following contexts that constrain only for the syntactic properties of incoming words, or to unrelated words in semantic priming paradigms where there is no need to access syntactic features at all) may be associated with increased activity within the left anterior superior temporal gyrus. While functional MRI lacks the temporal resolution necessary to evaluate this claim, consistent evidence comes from multimodal neuroimaging studies of both automatic and predictive semantic priming in healthy adults, which show that neural activity within the left anterior STG is rapidly suppressed when a target word follows a semantically related (versus unrelated) word (Lau et al., 2013, 2014).

Patients with schizophrenia exhibit abnormal functional connectivity between inferior frontal and temporal cortices during semantic processing tasks (Wolf et al., 2007; Griego et al., 2008; Li et al., 2010; Woodward et al., 2015). If, as we suggest above, these functional connections indeed form the neural substrate of the message-to-lexical generative circuitry, this disrupted functional connectivity in schizophrenia may be related to abnormalities in semantic and lexical processing in schizophrenia. On this account, an abnormally large semantic prediction error in schizophrenia should be associated with abnormally strong (i.e., non-attenuated) activity in temporal cortices in response to words that would normally be predictable in context. There is some evidence to support this hypothesis. During semantic priming, schizophrenia patients (Han et al., 2007; Kuperberg et al., 2007), as well as people at familial high risk for schizophrenia (Thermenos et al., 2013), show more activity within temporal cortices to semantically associated than unrelated word-pairs. Similar abnormal increases in activity within temporal cortices, together with abnormal decreases in frontal activity, are observed when patients engage in demanding semantic tasks such as deep semantic encoding, semantic retrieval (Kubicki et al., 2003; Ragland et al., 2004), and the comprehension of semantically implausible sentences (Kuperberg et al., 2008).

We know even less about how a breakdown of this neural circuitry might impact speech production in schizophrenia, leading to positive thought disorder. However, it is worth noting that severity of thought disorder in patients is negatively correlated with both the volume of left superior temporal gyrus (Shenton et al., 1992; Rajarethinam et al., 2000) and with activity of the left superior temporal gyrus during speech production (McGuire et al., 1998; Kircher et al., 2001), but positively correlated with activity in anterior inferior temporal cortices (McGuire et al., 1998). In healthy individuals, the production of speech is associated with *less* activity within the left anterior superior temporal cortex compared to the *pauses* in between clauses, perhaps because wordform-semantic (lexical) activity to self-produced words is largely “explained away” by prior predictions generated during speech planning. This difference is not seen in thought-disordered patients (Matsumoto et al., 2013), perhaps because patients fail to attenuate prediction error signals within inferior temporal cortices to self-produced words.

### The Generative Circuit Linking Higher-level Linguistic Representations and Low-level Perception

There is also evidence for abnormalities in the structure and function of brain regions involved in the perceptual processing of language in schizophrenia. For example, abnormalities around the left superior temporal gyrus, including primary auditory cortex, are well documented (e.g., Pearlson, 1997; Shenton et al., 2001; Steen et al., 2006; Sun et al., 2009). These abnormalities in low-level sensory cortex, together with the abnormalities described above, may contribute to disrupted generative circuits linking higher-level language processing with speech perception.

In healthy adults, predictions that are generated at relatively high levels of linguistic representation are propagated all the way down to sensory cortex, attenuating neural responses to expected stimuli. For example, *contra* the view that primary auditory cortex merely detects and relays information about acoustic features to higher-level association areas, different interpretations of the same ambiguous speech sound are associated with distinct neural signals in primary auditory cortex (Kilian-Hütten et al., 2011). In addition, the ability of auditory cortex to track and entrain to speech rhythm is stronger for intelligible speech (whose rhythmic properties are to an extent predictable from linguistic context) than for unintelligible speech (Peelle et al., 2013). Other studies have manipulated predictability more explicitly, finding, for example, that the neural response within the superior temporal gyrus is attenuated when spoken words match (versus mismatch) the content of prior written text (Sohoglu et al., 2012). Likewise, in the visual domain, syntactically unexpected printed words elicit stronger activation within occipital cortex than syntactically expected words, particularly when a syntactically unexpected word has low-level visual form features that are atypical of the expected syntactic category (Dikker et al., 2010).

Taken together, the findings described above suggest that, in healthy individuals, neural activity within perceptual regions is mainly evoked by stimuli that are unexpected rather than expected. There is some evidence suggesting that this may not be true in patients with schizophrenia. For example, the repetition of syllables under noisy conditions elicits stronger (i.e., less attenuated) neural responses in primary auditory cortex in patients than in healthy adults (Dale et al., 2009). In patients, reductions in the volume of the superior temporal gyrus (e.g., Delisi et al., 1994; Kasai et al., 2003; Chance et al., 2004) are associated with weaker prediction error signals: relatively more attenuation of neural responses to deviant auditory stimuli (McCarley et al., 1993).

Many of the studies investigating the structure and function of perceptual cortices in schizophrenia have subdivided patients according to whether they experience AVHs. Most such studies have found more pronounced structural and functional abnormalities in patients with hallucinations (although see, e.g., Woodruff et al., 1997). AVHs are correlated, for example, with the extent of volumetric reduction in superior temporal gyrus, including Heschl’s gyrus (e.g., Levitan et al., 1999; Rajarethinam et al., 2000; Gaser et al., 2004; Sumich et al., 2005). In addition, as outlined below, there is evidence that abnormal modulation of temporal cortices as well as abnormal frontotemporal functional connectivity are particularly pronounced in patients with hallucinations (Ford et al., 2002; Mechelli et al., 2007; Hashimoto et al., 2010; Oertel et al., 2010).

First, patients who experience AVHs exhibit abnormal patterns of functional connectivity and neural synchrony between left Heschl’s gyrus and surrounding brain regions involved in semantic and lexical language processing, memory, and other higher-level cognitive functions (e.g., Ford et al., 2002; Sommer et al., 2012; Shinn et al., 2013; de la Iglesia-Vaya et al., 2014). Second, whereas patients without AVHs and healthy adults exhibit stronger effective connectivity between left superior temporal regions and anterior cingulate cortex when hearing speech from another speaker (versus their own pre-recorded speech), patients with AVHs instead exhibit stronger effective connectivity between these regions when hearing their own (versus another speaker’s) voice (Mechelli et al., 2007). Third, when hearing speech stimuli that vary in predictability, patients actively experiencing AVHs generate weaker prediction error signals in primary auditory cortex to unpredictable stimuli (as well as omissions of expected stimuli) than healthy adults, and the magnitude of these prediction error signals is negatively correlated with neural signatures of AVHs within auditory cortex (Horga et al., 2014). Finally, there is some evidence that these weaker prediction error signals may be related to fronto-temporal dysconnectivity: ERP studies of the mismatch negativity (MMN) indicate that patients with AVHs, unlike patients without AVHs or healthy adults, show no significant differences in MMN amplitudes to speech deviants at frontal sites, and instead exhibit MMN effects primarily at temporal sites (Fisher et al., 2008). Taken together, these findings suggest links between abnormalities in the structure and connectivity of auditory cortex, abnormal prediction error, and the phenomenology of AVHs.

## Implications for Psychosocial Function and Cognitive Remediation

Thus far, we have primarily focused on the theoretical implications of characterizing perceptual, cognitive, and symptomatic sequelae of schizophrenia as consequences of disrupted generative models. We turn now to discussing how this perspective on language abnormalities might influence our understanding of real-world communicative dysfunction in schizophrenia, as well as the targeted development of cognitive and psychosocial remediation techniques to address such dysfunction.

### Psychosocial Communicative Function

When most clinicians think of language dysfunction in schizophrenia, they tend to focus on the symptoms of thought disorder and AVHs — the aspects of language dysfunction that are most easily diagnosed and characterized from a clinical perspective. As we have explained in this review, schizophrenia is associated with multiple pervasive abnormalities in both the higher- and lower-order aspects of language processing, and this is true not just of patients with thought disorder and AVHs, but also of patients without these overt symptoms. Such disturbances of language processing may not necessarily be detected on standard clinical interviews, which generally rely on fairly simple prompts and interchanges. Nor would they necessarily be detected on standard neuropsychological tools used to evaluate schizophrenia patients, which do not generally include measures that probe language comprehension. Importantly, however, such abnormalities in language processing are still likely to contribute considerably to psychosocial dysfunction in schizophrenia. We know, for example, that verbal memory performance predicts success in various forms of verbal therapy (Smith et al., 1999) and is associated with social, adaptive, and occupational success (Green et al., 2000; Holshausen et al., 2014). And, as discussed further below, many of the components or outcome measures of psychosocial and cognitive intervention programs are actually linguistic in nature. These include, for example, measures of conversational turn-taking skills, which are part of assessments of psychosocial skills and abilities (Hooley, 2010), and measures of patients’ ability to carry out actions based on spoken instructions, which are part of perceptual and cognitive training programs (e.g., Adcock et al., 2009; Fisher et al., 2009, 2015).

Language processing abnormalities, stemming from dysfunction of generative circuitry, are particularly likely to compromise real-world social communicative function in schizophrenia for at least three related reasons. First, in real-world communicative situations, we rarely hear individual words or speech sounds in isolation. Language is instead typically presented in phrases, sentences, text, and conversation, and is therefore highly context-dependent. As discussed above, predictions based on this high-level context are critical for us to disambiguate individual words and the low-level perceptual features of speech. Thus, problems that patients have in making higher-order causal inferences about the sources of linguistic signals (i.e., communicative intentions) and using these inferences predictively to accurately interpret incoming sensory data would likely be exacerbated in real-world communicative situations.

Second, in typical everyday communication, linguistic information does not unfold one sentence at a time with multi-second gaps in between utterances, as it does in many lab experiments. Rather, spoken language proceeds very quickly (approximately 5–8 syllables per second; Pellegrino et al., 2007) at a pace that is largely outside the listener’s control. Thus, any impairments in mobilizing higher-order representations to predict upcoming input would also likely be exacerbated in real-world communication. Moreover, while patients’ reliance on reactive feed-forward processing mechanisms might allow them to compensate and perform adequately on simple comprehension tasks, it would lead to considerable difficulties in interactive conversation, which requires individuals not only to produce and comprehend language in real time, but also to rapidly coordinate complex sequences of conversational turns with their interlocutors (e.g., Schegloff, 2000; Clark and Krych, 2004; de Ruiter et al., 2006; Stivers et al., 2009).

Third, in real-world communication, we must constantly and flexibly *adapt* language comprehension and production to the ever-changing demands of our social environment. As discussed throughout this review, we are able to use the discrepancies between our predictions and the actual input – as conveyed through prediction error signals – to adjust or switch to internal generative models of our conversational partners. In this way, we quickly adapt to how different speakers’ acoustic features and semantic and syntactic preferences cluster together, and adjust what we say in response to accommodate their preferences and expectations (Kleinschmidt and Jaeger, 2015). In schizophrenia, any failure to anticipate and compensate for contextually conditioned variation in language input will put patients at a processing disadvantage. This may be related to the cognitive rigidity and perseveration that can characterize language in schizophrenia (Cameron, 1944; Cohen, 1976; Andreasen, 1979a; Manschreck et al., 1985; Barr et al., 1989), and that may contribute to patients’ impairments in adapting to different social situations (Harrow et al., 1989).

These three factors highlight the importance of research that focuses on patients’ capacities in a wider range of real-world communicative contexts and that examines the mechanisms of comprehension breakdown in situations that more closely approximate real-world language use, such as interactive communication. This work could perhaps derive inspiration from elegant psycholinguistic paradigms designed to investigate interactive communication at multiple levels of representation in naturalistic contexts without sacrificing experimental control (e.g., Clark, 1992; Keysar et al., 2000; Sedivy, 2003; Clark and Krych, 2004; Brown-Schmidt and Tanenhaus, 2006; Brown-Schmidt and Konopka, 2011). In addition, our hypothesis that time-sensitive predictive language processing is particularly compromised in patients with schizophrenia makes it especially important to use techniques that give insights into the time course of processing, such as eye tracking, ERPs, and EEG/MEG. Comparing measures of real-time processing with responses elicited from patients in the absence of time pressure on the same tasks could shed further light on processing routes that may be relatively spared and could be targeted for cognitive remediation and prevention, as discussed below.

### Intervention Strategies

The theoretical perspective we offer here has important implications for psychosocial and cognitive rehabilitation strategies, which seek to increase patients’ quality of life both directly and indirectly via links between cognitive or perceptual function and psychosocial or everyday functional skills (Wykes and Huddy, 2009). A wide variety of cognitive remediation approaches are currently used in schizophrenia. They can be broadly divided into three classes of approaches.

The first involves real-world *psychosocial skills training*, which aims to support patients’ skills in both interpreting and responding to social situations (e.g., Wallace and Liberman, 1985; Benton and Schroeder, 1990; Wallace et al., 1992; Scott and Dixon, 1995; Heinssen et al., 2000; Bellack, 2004). Psychosocial skills training includes methods ranging from explicit skills instruction and coaching to the modeling and rehearsal of target behaviors in unstructured, structured, and controlled (via confederate) interactions. Because communication is central to social interaction, many of these target behaviors are verbal, with a primary focus at the level of inferring and expressing communicative intentions. This type of training generally targets patients’ performance within specific functional domains, and its effectiveness has not been found to generalize to other domains. On the other hand, any effects that communicative skills training might have on more general language communicative abilities remain largely unassessed.

A second class of approaches focuses on *high-level cognitive training*, which aims to improve cognitive or meta-cognitive abilities, such as working memory, problem solving, attention, or learning styles (reviewed by Medalia and Choi, 2009). This approach is motivated by evidence for links between cognitive dysfunction and psychosocial dysfunction in schizophrenia (e.g., Breier et al., 1991; Bowie et al., 2010). Cognitive training approaches do not target language skills directly, but instead target more “domain-general” cognitive functions, which are sometimes assumed to underlie language abilities. For example, problems in using high-level context during language comprehension may be compounded by or even reducible to problems with maintaining message-level representations within working memory and/or impairments in domain-general “cognitive control” mechanisms (e.g., Cohen and Servan-Schreiber, 1992; Boudewyn et al., 2012), although the extent to which this is the case is open to debate (for discussion, see Kuperberg, 2010b). The core assumption of cognitive training approaches then, is that improving domain-general cognitive abilities like working memory and cognitive control should lead to improved real-world psychosocial functioning, including communicative functioning. And indeed, meta-analyses of the effectiveness of cognitive remediation techniques generally find some degree of transfer from specifically trained domains to more general measures of daily functioning (e.g., Medalia and Choi, 2009; Wykes et al., 2011).

Finally, a third set of approaches focuses instead on *low-level perceptual training* tasks (e.g., Adcock et al., 2009; Fisher et al., 2009, 2015; Norton et al., 2011; Popov et al., 2011; Biagianti and Vinogradov, 2013). These tasks gradually increase in complexity over the course of training. Notably, many of them use auditory stimuli and have clear relevance for language processing. For example, auditory perceptual training includes tasks ranging from basic perceptual discrimination of auditory stimuli with rising or falling pitch, to more complex verbal tasks, like performing actions based on spoken instructions (Adcock et al., 2009; Fisher et al., 2009, 2015). The assumption of these types of programs is that targeting low-level perceptual representations enhances the quality of sensory information available to higher-level cognitive operations such as language comprehension, and that this will therefore gradually improve overall cognitive and functional outcomes. This assumption is shared by theoretical views that sensory abnormalities in schizophrenia are the root cause of upstream disruptions to higher-order representations and processes (Javitt, 2009; Javitt and Freedman, 2015). Evaluations of perceptual training techniques have generally found some degree of transfer to more global measures of cognition (e.g., Fisher et al., 2009, 2015), though it is less clear whether gains translate to functional outcomes.

Despite their different approaches, these three types of remediation programs – those that target psychosocial skills, high-level cognitive processes, and low-level sensory processing – all yield moderate to strong gains on performance within targeted domains (e.g., Heinssen et al., 2000; Bellack, 2004; Reddy et al., 2014). However, the mechanisms underlying the success of cognitive and perceptual remediation programs, both within and across domains, are not well understood. Responsiveness to remediation is heterogeneous across patients, and few factors have been identified that predict whether an individual will benefit from remediation (Medalia and Choi, 2009). We suggest that the similarities in efficacy across different programs of remediation is consistent with the idea that the core dysfunction in schizophrenia is not specific to individual perceptual or cognitive domains, but instead lies in mechanisms that *link* these domains. We further suggest that these cross-domain mechanisms involve the breakdown of generative circuits linking higher-level cognitive representations and processes to lower-level perception.

Viewing cognitive remediation through the lens of generative models makes a further prediction: that the *combination* of high- and low-level approaches might actually have synergistic rather than additive effects on overall cognitive and perceptual functioning. Targeting high-level processes and representations using high-level cognitive training approaches should improve functioning across levels by supporting patients’ internal models of context and their ability to link prior knowledge to incoming stimuli. Conversely, improving patients’ sensory representations using lower-level perceptual training approaches should improve functioning at higher levels of representation by decreasing uncompensated prediction error throughout the generative model. Supporting processing at multiple levels of representation in tandem may therefore make it easier to remedy the self-reinforcing cycle of disrupted prediction and uncompensated prediction error than targeting either end of this cycle in isolation.

The development of comprehensive combined remediation programs may also hold promise for linking perceptual and cognitive gains to higher-order behavioral, social, and occupational function. There is already some evidence that is consistent with this idea. The most effective strategy for transferring neurocognitive gains associated with high-level cognitive remediation to improvements in real-world behaviors and functioning has been to pair high-level cognitive remediation with psychosocial therapy or functional skills training (e.g., McGurk et al., 2005; Bowie et al., 2012; Reddy et al., 2014). Again, the mechanisms that support transfer between these domains are not well understood. However, these observations provide additional support for the utility of viewing language as an integrated system of perception, cognition, and action, in which abstract behavioral and social goals are integrally linked to cognitive processes and low-level perception.

Finally, it may be fruitful to explore the efficacy of a combined remediation approach for preventing or mitigating the onset of psychosis in individuals at high risk for developing schizophrenia. Both language abnormalities (e.g., Fuller et al., 2002; Simon et al., 2007; Wood et al., 2007; Solomon et al., 2011; Thompson et al., 2011) and social difficulties (Hans et al., 2000; Cornblatt et al., 2007) are detectable prior to the onset of the illness, possibly reflecting some link in their developmental trajectory. Indeed, the generative perspective suggests that the inferential, predictive, and adaptive processes that we engage in typical adult language processing are in fact the same processes involved in language learning and development. Extended to schizophrenia, this perspective connects with recent work discussing neurodevelopmental aspects of schizophrenia in relation to CNS development throughout the lifespan (Nour and Howes, 2015). More generally, the generative perspective on language abnormalities in schizophrenia strongly suggests that targeting psychosocial, cognitive, and perceptual abilities early in the course of schizophrenia may provide the best window of opportunity for bringing the vicious cycle of abnormal prediction and belief updating back under control.

## Conflict of Interest Statement

The authors declare that the research was conducted in the absence of any commercial or financial relationships that could be construed as a potential conflict of interest.

## Footnotes

1 Most traditional models do acknowledge an important role of prediction in ensuring efficient and reliable comprehension. However, it is not seen as a driving force behind comprehension itself.

2 Classical frameworks of language production also acknowledge an important role of monitoring – both internal monitoring (e.g., Levelt, 1983; Postma, 2000; Hartsuiker and Kolk, 2001) and external monitoring of one’s interlocutor for comprehension (e.g., Clark and Krych, 2004). In many of these models, however, monitoring is conceptualized as a separate ‘add-on’ mechanism designed to minimize speech errors, that is taken over by either the comprehension system (Levelt, 1983) or by a distinct executive mechanism (but see Dell, 1986, 2013 for discussions of how loops of cascading forward and feedback activation between units at adjacent levels of the system may function to instantiate such monitoring). Within a generative architecture, monitoring arises as an inevitable consequence of the overarching goal of minimizing overall prediction error in transmitting a message-level representation from one mind to another (Jaeger and Ferreira, 2013; Jaeger and Snider, 2013).

3 More generally, it has been proposed that top-down predictions may play this type of role in facilitating action, e.g., by facilitating reflexive movements that effectively correct prediction errors and ensure that action outcomes approximate what is predicted (Adams et al., 2013a; Brown et al., 2013).

4 Early studies of lexical ambiguity resolution suggested that effects of context on homophone interpretation were delayed relative to the initial retrieval of potential alternative meanings (Swinney, 1979; Tanenhaus et al., 1979). However, subsequent studies using more sensitive on-line measures and fine-grained manipulations have qualified this finding, suggesting complex interactions between multiple graded factors such as strength of contextual constraint, relative frequency of alternative interpretations, and timing (e.g., Duffy et al., 1988; Li and Yip, 1998; Dixon and Twilley, 1999; Calacouris and Brock, 2009), such that context appears to have delayed effects only when all available sources of evidence, taken together, do not strongly favor one alternative over another.

5 Some researchers have characterized this problem as a failure to suppress activated lexical representations that are incongruous with sentence context (e.g., Titone et al., 2000). This possibility is more in keeping with traditional models of language comprehension positing that the incoming language signal activates successively higher-level or more abstract linguistic representations primarily from the bottom-up, and that context acts to constrain interpretation at a later stage of processing (e.g., Swinney, 1979; Tanenhaus et al., 1979). That is, multiple possible words are first activated, and contextually inappropriate or less likely words are then suppressed. Our proposal, in contrast, is that, by informing our inferences and predictions, contextual information normally constrains the set of alternatives that are considered, even prior to encountering the incoming language signal, and that it is a breakdown of these generative processes that gives rise to indiscriminate activation of semantic neighborhoods in schizophrenia.

6 Semantic priming under more controlled strategic conditions, however, is generally *reduced* in schizophrenia patients, both with and without thought disorder (reviewed in Kuperberg et al., 2008), perhaps as a result of the breakdown of generative circuits that link lexical representations with other higher-level representations, such as those describing semantic relationships across words.

7 Although most of the research described in this section pertains to the auditory processing of speech, the principles and concepts described in this section also extend to the visual processing of written language.

8 The activation of these representations may or may not consciously be experienced as “inner speech” – the internal monologue? that some individuals experience on a regular basis, and that we generally correctly perceive as happening inside our own heads (e.g., Vygotsky, 1987).

9 It has been proposed that an analogous breakdown of agency over our own “thought” accounts for delusions (Currie, 2000; Currie and Jureidini, 2001). While the representations underlying thought and their relationship to the representations underlying language remain a topic of philosophical debate, one proposal is that propositional thought is mediated by a complex combinatorial semantic system (Jackendoff, 2002; Pinker, 2007; see also Hinzen, 2013). This may include metaphorical and metonymic models that allow us to map certain abstract concepts on to other domains, e.g., being scared or angry mapping to feeling possessed by devils (Lakoff, 1971; Lakoff and Johnson, 2008). A loss of agency over such a system might, for example, lead a thought like, “I am possessed by devils”, to be perceived as being possessed by devils that originate in the real world (Rhodes and Jakes, 2004; Fineberg et al., 2015b), or a thought like, “I am like Jesus”, to be perceived as the real-world state, “I am Jesus” (Hinzen, 2015). Similarly, transitory thoughts about innocuous real-world events may also lead patients to perceive such events as highly salient and self-relevant (Kapur, 2003; Fletcher and Frith, 2009). Within the generative framework proposed here, this loss of agency may arise from a breakdown of the circuitry that links thought with underlying interpretations or beliefs about real-world events/states. A failure to explain away or attenuate prediction error at the level of thought (see also Fletcher and Frith, 2009; Corlett et al., 2010) might lead to patients’ subjective conviction that their own thoughts stem from real-world external events or states, even though, unlike true beliefs, there is often little attempt to justify them based on objective evidence (Berrios, 1991; Currie and Jureidini, 2001; Hinzen, 2015).
